# Stocking density-induced changes in growth performance, blood parameters, meat quality traits, and welfare of broiler chickens reared under semi-arid subtropical conditions

**DOI:** 10.1371/journal.pone.0275811

**Published:** 2022-10-13

**Authors:** Kwena Kgaogelo Thema, Caven Mguvane Mnisi, Victor Mlambo

**Affiliations:** 1 Department of Animal Science, Faculty of Natural and Agricultural Science, North-West University, Mafikeng, South Africa; 2 Food Security and Safety Focus Area, Faculty of Natural and Agricultural Science, North-West University, Mafikeng, South Africa; 3 School of Agricultural Sciences, Faculty of Agriculture and Natural Sciences, University of Mpumalanga, Nelspruit, South Africa; University of Agriculture Faisalabad, PAKISTAN

## Abstract

Broiler production in semi-arid tropics must contend with high levels of heat stress, which have implications on stocking density, bird welfare, and profitability. Under these conditions, optimal stocking densities are likely to be lower than expected, thus must be experimentally determined. Therefore, this study investigated growth performance, haematology, serum biochemistry, carcass and meat quality, sizes of internal organs, and stress biomarkers in response to different stocking densities in broilers reared under semi-arid subtropical conditions. Five hundred, day-old Ross 308 broilers (44.0 ± 5.24 g live-weights) were randomly distributed to 25 replicate pens (1.32 m^2^ floor space each) to create five stocking densities: 1) 10 birds/pen (SD10); 2) 15 birds/pen (SD15); 3) 20 birds/pen (SD20); 4) 25 birds/pen (SD25); and 5) 30 birds/pen (SD30). There was a linear decrease (*P* < 0.05) in overall feed intake and weight gain in weeks 2 and 3 as stocking density increased. However, weight gain showed positive and negative quadratic responses (*P* < 0.05) in weeks 5 and 6, respectively, as stocking density increased. No linear or quadratic effects (*P* ˃ 0.05) were observed for overall feed conversion ratio, haematological parameters, and meat quality traits in response to stocking density. Symmetric dimethylarginine, alanine transaminase, and albumin levels quadratically increased (*P* < 0.05) in response to increasing stocking densities. Serum glucose and thigh weight were not affected (*P* < 0.05) while final body, drumstick, breast, and wing weights linearly declined with stocking density. Increasing stocking density linearly reduced (*P* < 0.05) the weights of gizzard, proventriculus, caecum, and colon. Stocking density had no effect (*P* ˃ 0.05) on latency-to-lie. It was concluded that higher stocking densities compromised feed intake, resulting in poor weight gains. Based on weight gain trends observed in week 5, it was determined that Ross 308 broilers should be reared at no more than 20 birds/pen (~15 birds/m^2^ or 27.27 kg/m^2^) under the experimental ambient conditions compared to the much higher globally accepted industry standard of 20 birds/m^2^.

## Introduction

Broiler chickens require favourable rearing conditions to maximise their genetic potential for growth. Significant deviations from optimum rearing conditions can compromise feed utilization, growth performance, and bird welfare [1]. Stocking density is one critical rearing factor that has serious implications on the economic and social sustainability of the poultry industry. Globally, the accepted industry standard is to achieve between 30 and 38 kg bodyweight per m^2^ or to produce 20 adult birds (35 d of age) per m^2^ [2]. However, broiler producers often resort to high stocking densities to increase profit margins per unit area [3], despite the documented negative effects of this practice on bird performance [4, 5]. High stocking densities are especially detrimental to birds during the finisher phase when the bodyweight per unit area ratio is very high. Many other studies have demonstrated that high stocking density reduces growth and slaughter weight at day 42 [6–8]. Uzum & Toplu [9] and Das & Lacin [10] also reported negative effects of high stocking density (18 and 20 birds/m^2^) on overall feed intake, growth rates and feed conversion ratio in broilers from day 21. Less reported negative effects of high stocking densities include high ammonia and litter moisture levels, increased incidences of foot pad lesions, reduced preening behaviour, high heat stress and reduced locomotion in broiler chickens [11–14]. Nahashon et al. [15] reported that high stocking density rates have a negative effect on overall carcass performance of French guinea broilers. Moreover, Simitzis et al. [8] also observed a significant decrease in intramuscular fat due to higher stocking density (27.2 kg/m^2^). Accordingly, exceeding optimum (recommended) stocking densities has debilitating effects on bird welfare and profitability of the enterprise. On the other hand, using stocking densities below optimum (recommended) levels also has negative impacts on profitability due to underutilization of space. Therefore, there is a need to carefully determine functional stocking densities for different production systems and climatic conditions. Unlike the association between stocking density and farm profitability, which is rather straightforward, the relationship between stocking density and welfare is much more complex and difficult to unravel. Although many authors have studied this relationship, different results have been reported. This could be attributed to differences in production (experimental) systems, accuracy and validity of indicators of welfare, and climatic conditions.

While there are generally accepted industry standards regarding stocking density for broiler production, these are likely to vary depending on factors such as broiler strain, husbandry systems, and climatic conditions. For example, in some semi-arid subtropical areas, high temperatures and low humidity modulate stocking density effects on bird productivity and welfare. In such areas, it is imperative that optimal stocking density be experimentally determined. Thus, the aim of this study was to determine the optimal stocking density for Ross 308 broilers reared in a semi-arid subtropical environment using growth performance, blood and meat quality parameters, and welfare indicators. We hypothesized that high stocking densities would compromise the physiology, meat quality, and welfare indicators of the birds and that the semi-arid subtropical rearing conditions require a much lower stocking density than the industry standard of 20 birds/m^2^.

## Material and methods

### Feeding trial

The procedures employed during the rearing, handling, and slaughtering of the chickens were approved (NWU-02006-20-A5) by the Research Ethics Committee for Animal Production studies at the North-West University (Mafikeng, South Africa). The six-week feeding trial was conducted during the autumn season at the North-West University Experiential Farm (26º41’36” S, 27º 05’35” E). The temperature ranged between a minimum of 12°C at night and a maximum of 30°C during the day. Five hundred, day-old male Ross 308 chicks were bought from Superbird (Pty) Ltd (Brits, South Africa), weighed, and randomly distributed to 25 replicate pens (experimental units), each measuring 1.1 m L × 1.2 m W × 1.55 m H, providing a floor space of 1.32 m^2^, including space occupied by feeders and drinkers. Standing pens (partitioned into two), built using wire-mesh, were used for this study and polyethene plastics were used to cover the floors. In a completely randomised design, the chicks were reared under five different stocking densities as follows: 10 birds/pen (SD10), 15 birds/pen (SD15), 20 birds/pen (SD20), 25 birds/pen (SD25) and 30 birds/pen (SD30). The recommended market weight for broilers in South Africa is currently 1.8 kg, therefore, these experimental stocking densities can be translated into SD10 = 13.64 kg/m^2^, SD15 = 20.45 kg/m^2^, SD20 = 27.27 kg/m^2^, SD25 = 34.09 kg/m^2^ and SD30 = 40.91 kg/m^2^. Each stocking density group was replicated five times. The birds were reared on starter (0–21 days), grower (22–35 days) and finisher (36–42 days) commercial diets (Table 1), which were bought from Nutrifeeds (Lichtenburg, South Africa). The chemical composition and digestible amino acids in starter, grower, and finisher diets used for feeding the broilers are presented in S1 Table. For the entire duration of the feeding trial, the commercial diets and clean, fresh water were offered *ad libitum* to the birds.

**Table 1:** Ingredient composition (%, as fed basis) of experimental starter, grower, and finisher phase diets.

Ingredients	Starter phase	Grower phase	Finisher phase
Maize	42.71	46.37	51.51
Soy oilcake	16.0	16.0	13.0
Sunflower oilcake	5.0	0	0
Wheat bran	32.5	34.5	32.5
Limestone	1.1	1.0	0.95
Mono-calcium phosphate	1.1	0.95	0.8
Phytase	0.01	0.01	0.01
Salt	0.15	0.2	0.2
Methionine	0.29	0.24	0.22
Threonine	0.15	0.15	0.1
Lysine	0.39	0.28	0.26
Vitamin mix	0.25	0.1	0.2
Zinc-Bacitracin	0.05	0.05	0.05
Monensin 20%	0.05	0.05	0.05
Sodium bicarbonate	0.03	0.2	0.2

### Performance measurements

Feed intake (FI) was measured daily by calculating the difference between feed offered and feed refusals. All the birds in every pen were weighed on a weekly basis by weighing them on scale to calculate average weekly body weight gain (ABWG). Feed conversion efficiency (FCE) was determined as weight gain divided by feed intake.

### Blood collection and analysis

On the final day (day 42), two birds were randomly selected from each pen and blood samples were collected from the brachial vein. A needle (23-gauge) and syringes (5 mL) were used to collect blood and immediately the blood was transferred into serum and whole blood tubes. An automated LaserCyte Haematology Analyzer (IDEXX Laboratories Inc., Gauteng, South Africa) was used to determine white blood cells, lymphocytes, haematocrit, monocytes, heterophils, and platelets. An automated Vet Test Chemistry Analyzer (IDEXX Laboratories Inc., Gauteng, South Africa) was used to analyse for total protein, gamma-glutamyl transferase (GGT), glucose, albumin, cholesterol, symmetric dimethylarginine (SDMA), creatinine, phosphorus, alanine transaminase (ALT), alkaline phosphatase (ALKP), calcium and urea.

### Carcass traits and internal organs

At day 42 of age, all the birds were weighed to calculate final body weight (FBW). Immediately after weighing, all the birds were transported to a locally registered abattoir. Firstly, the birds were electrically stunned and slaughtered by cutting the jugular vein with a sharp knife. After bleeding, the plucker machine was used to remove all feathers before the carcasses were manually eviscerated. Records of hot carcass weight (HCW) were immediately taken after slaughter. Carcass yield was calculated by expressing HCW as a proportion of slaughter weight (FBW). Weights of drumstick, breast, thigh, wing, liver, gizzard, spleen, heart, and proventriculus were measured. The duodenum, jejunum, ileum, caecum, and colon with their contents were weighed and expressed in grams.

### Meat quality measurements

A meat pH meter (HI98163, Hanna Instruments, Woonsocket, RI, USA) was used to determine the pH of breast meat after slaughter. The pH meter was calibrated with pH 4, 7, and 10 standard solutions, provided by the supplier, after taking measurements from every replicate pen. Meat lightness (L*), yellowness (b*) and redness (a*) were measured on the surface of the breast muscle using a Minolta colour spectrophotometer (BYK-Gardener GmbH, Geretsried, Germany), which has a 20-mm diameter measurement area (aperture size) and an illuminant D65-day light. Measurements were collected using a 10° observation angle. The colour guide was calibrated and set following the manufacturer’s recommendation. Cooking loss was determined by pre-weighing raw breast muscle and then placed in a foil plate and cooked to reach 75°C internal temperature as described by Honikel at al. [16]. Another set of raw breast meat samples were sheared using a Meullenet-Owens Razor Shear Blade (A/MORS) mounted on a Texture Analyzer (TA.XT plus, Stable Micro Systems, Surrey, UK) to determine shear force (N). Breast meat water holding capacity (WHC) was determined following the filter-paper press method by Grau and Hamm [17]. Drip loss was determined by suspending 80–120 g pieces of breast muscle in closed containers, allowing them to drip. The samples were left in a chilled environment (1–5°C) for 72 hours before re-weighing to calculate the percentage of fluid lost due to dripping as described by Honikel at al. [16].

### Latency-to-lie

At day 42, two birds per pen were randomly selected for the latency-to-lie test (LTL). The LTL as defined by Berg and Sanotra [18], focuses on the body interaction with water, as it is an unusual experience for broiler chickens. All the participating birds were individually placed in a plastic container filled with 3 cm of water at 32°C. The time it took for the bird to lie down and come into contact with the water was recorded. This test is based on the principle that the longer the bird stays on its feet before lying down (touching the water), the better is its leg health. If the bird was still standing after 10 minutes, the test was terminated, and the legs judged to be strong and healthy.

### Statistical analysis

Measurements from multiple broilers per pen were averaged before analysis, resulting in each replicate pen having one average value per parameter of interest. Data were analysed for homogeneity of variance and for normality using Levene’s test and the NORMAL option in the Procedure Univariate statement, respectively. Weekly feed intake (FI), weight gain and feed conversion efficiency (FCE) data were analysed using the repeated measures in the general linear model (GLM) procedure of SAS [19]. Response surface regression analysis (Proc RSREG; SAS [19]) was employed to determine linear and quadratic effects of different stocking density rates. The optimum stocking density (*y = -b/2a*) was estimated from significant quadratic responses. Overall growth performance, blood parameters, carcass characteristics, internal organs, meat quality and welfare indicators data were analysed using the GLM procedure of SAS, where stocking density was the only factor. For all measured parameters, significance was declared at *P <* 0.05 and least squares means were compared using the probability of difference option.

## Results

Repeated measures analysis revealed significant week × stocking density interaction effect on average weight gain (*P* = 0.019), but not on FI (*P* = 0.184) and FCE (*P* = 0.072). Table 2 shows that overall feed intake linearly declined [*y* = 4890 (±0.014)– 98.7 (±45.26) *x*; R^2^ = 0.45, *P* = 0.0002] as stocking density increased. Neither linear nor quadratic effects were observed for overall FCE as stocking density increased. Average weight gain declined in week 1 [*y* = 100.7 (±16.96)– 2.61 (±1.84) *x*; R^2^ = 0.16, *P* = 0.046] and week 2 [*y* = 296.7 (±44.80)– 4.58 (±4.87) *x*; R^2^ = 0.26, *P* = 0.01] in response to increasing stocking density. In week 5, a positive quadratic effect was observed for average weight gain [*y* = 967.8 (±209.9) + 60.07 (±28.39) *x*– 1.57 (±0.70) *x*^2^; R^2^ = 0.18, *P* = 0.03], but in week 6 a negative quadratic effect [*y* = 967.8 (±209.91)– 53.21 (±22.83) *x* + 1.28 (±0.56) *x*^2^; R^2^ = 0.18, *P* = 0.03] was observed. From the positive quadratic weight gain response in week 5, an optimum stocking density of 20 birds/pen (~27.27 kg/m^2^) was determined. There were no stocking density effects on overall BWG and FCE but overall FI was significantly influenced. The SD10 group had higher overall FI (4096.2 g/bird) than the SD20, SD25 and SD30 groups, which did not differ (*P* > 0.05).

**Table 2:** The effects of increasing stocking density on growth performance of Ross 308 broiler chickens.

	^1^Stocking density rates						*P* value		
^2^Parameters	SD10	SD15	SD20	SD25	SD30	^3^SEM	GLM	Linear	Quadratic
Overall FI (g/bird)	4096.2^b^	3747.1^ab^	3649.9^a^	3505.7^a^	3483.6^a^	108.7	0.005	0.0001	0.135
Overall FCE	0.516	0.548	0.514	0.528	0.536	0.01	0.708	0.746	1.000
	**Average weekly weight gain (g/bird)**								
Week 1	76.25	82.26	61.73	67.55	69.33	3.543	0.952	0.046	0.274
Week 2	261.8	234.9	226.7	235.6	211.3	11.16	0.060	0.010	0.600
Week 3	353.4	292.6	271.3	256.3	285.8	30.59	0.248	0.077	0.101
Week 4	396.8	436.8	289.5	357.2	364.5	30.07	0.170	0.199	0.259
Week 5	478.47	492.5	651.2	513.1	401.1	65.24	0.125	0.525	0.035
Week 6	537.6	519.9	385.2	415.7	539.4	52.50	0.428	0.553	0.033

^a,b^Means with common superscripts do not differ (*P* > 0.05).

^1^Stocking density rates: SD10 = stocking density of 10 birds/pen; SD15 = stocking density of 15 birds/pen; SD20 = stocking density of 20 birds/pen; SD25 = stocking density of 25 birds/pen; SD30 = stocking density of 30 birds/pen.

^2^Parameters: FI = feed intake; FCE = feed conversion efficiency.

^3^SEM = standard error of the mean.

Table 3 reveals that there were no linear or quadratic effects (*P* > 0.05) for all haematological parameters in response to increasing stocking density in broilers. Quadratic effects were observed for SDMA [y = –5.30 (±2.57) + 1.03 (±0.27) *x–* 0.02 (±0.007) *x*^2^; R^2^ = 0.144, *P* = 0.002], albumin [*y* = –91.41 (±59.10) + 21.05 (±6.43) *x*– 0.047 (±0.16) *x*^2^; R^2^ = 0.236, *P* = 0.007] and ALT [*y* = –61.61 (±56.36) +17.12 (±6.13) *x*– 0.35 (±0.15) *x*^2^; R^2^ = 0.137, *P* = 0.030] as stocking density rates increased. Birds reared under SD10 had the lowest level of SDMA (2.7 μg/L), albumin (68.7 g/L), and ALT (69.3 g/L) compared to other stocking density groups. In contrast, birds raised under SD20 had the lowest GGT (31.68 U/L) while SD25 and SD30 birds had the highest GGT (36.28 U/L and 35.17 U/L, respectively).

**Table 3:** The effects of increasing stocking density on blood parameters of Ross 308 broiler chickens.

	^1^Stocking density rates						*P* value		
^2^Parameters	SD10	SD15	SD20	SD25	SD30	^3^SEM	GLM	Linear	Quadratic
Lymphocytes (×10^9^/L)	2.54	2.79	1.92	2.56	1.50	0.405	0.189	0.090	0.407
Monocytes (×10^9^/L)	1.24	1.31	2.56	1.45	1.81	0.464	0.353	0.403	0.426
Platelets (×10^9^/L)	47.26	47.33	39.66	38.95	35.74	8.051	0.792	0.210	0.986
Heterophils (×10^9^/L)	11.32	14.27	14.81	14.19	11.56	2.891	0.866	0.963	0.255
WBC (×10^9^/L)	15.26	18.41	19.29	18.23	14.91	3.186	0.828	0.927	0.216
Haematocrits (%)	36.34	34.3	34.25	36.4	36.0	0.828	0.209	0.605	0.124
Glucose (mmol/L)	6.22	5.98	6.05	5.77	5.63	0.217	0.356	0.047	0.856
Creatinine (mol/L)	9.42	10.1	9.5	9.6	9.3	0.645	0.919	0.711	0.595
SDMA	2.70^a^	4.77^c^	5.9^d^	5.99^e^	4.51^b^	0.677	0.017	0.027	0.003
Phosphorus (mmol/L)	4.25	3.72	5.25	4.5	4.67	0.512	0.34	0.344	0.662
Calcium (mmol/L)	2.36	2.38	2.19	2.38	2.35	0.087	0.506	0.944	0.406
Total protein (g/L)	105	109.7	111.7	108.9	107	5.434	0.922	0.847	0.364
Albumin (g/L)	68.7^a^	125.8^c^	142.4^e^	134.41^d^	121.73^b^	15.39	0.024	0.023	0.007
ALT	69.3^a^	125.8^b^	142.4^e^	134.41^c^	141.73^d^	14.33	0.009	0.002	0.03
ALKP	390.6	431.5	449.6	488.6	516.1	59.48	0.615	0.100	0.978
GGT	33.35^c^	32.17^b^	31.68^a^	36.28^e^	35.17^d^	1.118	0.04	0.056	0.262
Cholesterol (mmol/L)	4.59	4.34	4.27	4.58	4.95	0.384	0.749	0.417	0.251
Urea (mmol/L)	2.15	2.01	2.22	2.07	2.54	0.211	0.451	0.212	0.278

^a,b,c,d,e^Means with common superscripts do not differ (*P* > 0.05).

^1^Stocking density rates: SD10 = stocking density of 10 birds/pen; SD15 = stocking density of 15 birds/pen; SD20 = stocking density of 20 birds/pen; SD25 = stocking density of 25 birds/pen; SD30 = stocking density of 30 birds/pen.

^2^Parmeters: WBC = white blood cells; ALT = alanine transaminase; ALKP = alkaline phosphatase; SDMA = symmetric dimethylarginine; GGT = gamma-glutamyl transferase.

^3^SEM = standard error of the mean.

Table 4 shows that there were linear decreases (*P* < 0.05) for FBW, wing, drumstick, liver, spleen, heart, duodenum, jejunum, and ileum weights in response to increasing stocking density. Chickens on SD10 and SD15 had higher (*P* < 0.05) FBW than SD20. The SD20 group had the highest carcass yield (83.59 g) while the lowest was from the SD15 group (72.46 g). Birds reared on SD30 and SD25 groups had the least wing weight compared to other groups. The SD25 group had lower drumstick weight (87.28 g) compared to SD 20 (94.60 g), which was the highest. For duodenum weight, SD25 (16.38 g) and SD30 (16.92 g) birds had the least weights compared to other stocking density groups.

**Table 4:** The effects of increasing stocking density on carcass characteristics and internal organs (g, unless stated otherwise) of Ross 308 broiler chickens.

	^1^Stocking density rates						*P* value		
^2^Parameters	SD10	SD15	SD20	SD25	SD30	^3^SEM	GLM	Linear	Quadratic
FBW	2194^cb^	2324^c^	1866^a^	1902^ab^	1916^ab^	79.21	0.001	0.003	0.464
Carcass yield (%)	77.34^d^	72.46^a^	83.59^e^	76.67^c^	75.98^b^	2.27	0.044	0.861	0.350
HCW	1698.50	1664.6	1564.3	1459.3	1456.5	51.34	0.007	0.0003	0.763
Breast	362.6	386.9	369.3	335.8	335.2	15.16	0.109	0.039	0.265
Wing	84.19^cd^	87.62^d^	82.80^bc^	76.28^ab^	75.37^a^	2.55	0.012	0.002	0.300
Thigh	128.6	128.2	123.3	115.0	115.8	4.90	0.174	0.017	0.953
Drumstick	99.42^b^	98.91^b^	94.60^ab^	87.28^a^	88.48^ab^	2.83	0.015	0.001	0.968
Liver	39.17	40.39	36.80	36.38	35.83	1.28	0.090	0.016	0.941
Gizzard	30.22	28.90	29.75	28.59	27.87	0.89	0.385	0.083	0.805
Spleen	2.497	2.428	2.296	2.165	2.200	0.129	0.334	0.040	0.659
Proventriculus	8.448	7.699	7.867	7.857	7.701	0.26	0.269	0.119	0.311
Heart	12.39	12.34	11.70	10.86	11.47	0.47	0.167	0.037	0.533
Duodenum	18.16^ab^	18.65^b^	17.92^ab^	16.38^a^	16.92^a^	0.47	0.017	0.007	0.710
Jejunum	30.23	29.47	28.73	28.52	26.95	0.89	0.157	0.011	0.738
Ileum	26.43	24.44	22.81	21.43	21.92	1.33	0.091	0.007	0.290
Caecum	13.79	13.84	14.39	14.55	13.55	0.42	0.426	0.865	0.125
Colon	3.016	2.704	3.078	3.114	2.590	0.26	0.519	0.599	0.446

^a,b,c,d^Means with common superscripts do not differ (*P* > 0.05).

^1^Stocking density rates: SD10 = stocking density of 10 birds/pen; SD15 = stocking density of 15 birds/pen; SD20 = stocking density of 20 birds/pen; SD25 = stocking density of 25 birds/pen; SD30 = stocking density of 30 birds/pen.

^2^Parmeters: FBW = Final body weight; HCW = Hot carcass weight.

^3^SEM = standard error of the mean.

There were neither linear nor quadratic trends (*P* ˃ 0.05) for all meat quality parameters as stocking density increased (Table 5). The GLM analysis also showed no significant effect of stocking densities on measured meat quality traits of the birds.

**Table 5:** The effects of increasing stocking density rates on meat quality parameters of Ross 308 broiler chickens.

	^1^Stocking density rates						*P* value		
Parameters	SD10	SD15	SD20	SD25	SD30	^3^SEM	GLM	Linear	Quadratic
Cook loss (%)	15.27	17.84	14.61	17.08	14.82	1.838	0.653	0.779	0.568
Drip loss (%)	2.86	2.77	2.45	3.88	1.95	0.847	0.598	0.794	0.553
^2^WHC (%)	18.91	22.8	21.74	21.46	18.45	1.993	0.438	0.694	0.074
Shear force (*N*)	3.75	3.77	3.88	3.75	3.62	0.09	0.353	0.282	0.100
pH	5.84	5.98	6.06	5.75	5.87	0.07	0.054	0.529	0.167
L* (Lightness)	58.25	60.76	55.15	56.67	57.83	1.95	0.369	0.446	0.562
a* (Redness)	2.19	1.54	1.61	2.31	1.76	0.34	0.405	0.928	0.537
b* (Yellowness)	12.16	13.3	11.84	11.66	12.35	1.35	0.922	0.762	0.941
Chroma	12.38	13.39	11.97	11.93	12.49	1.35	0.940	0.765	0.919
Hue angle	1.39	1.45	1.43	1.37	1.42	0.03	0.248	0.847	0.551

^1^Stocking density rates: SD10 = stocking density of 10 birds/pen; SD15 = stocking density of 15 birds/pen; SD20 = stocking density of 20 birds/pen; SD25 = stocking density of 25 birds/pen; SD30 = stocking density of 10 birds/pen.

^2^WHC = water holding capacity.

^3^SEM = standard error of the mean.

There were neither quadratic nor linear effects (*P* ˃ 0.05) observed for latency-to-lie (LTL) as the stocking density increased. The recorded LTL ranged from 138.8 to 276.8 seconds.

## Discussion

Stocking density is a vital management factor in broiler production because it is directly related to bird welfare, productivity, and profitability [20]. While higher stocking densities may reduce labour, equipment, energy, and housing costs, exceeding optimum stocking quickly reverses these gains by compromising the health, welfare, and productivity of birds [20]. In addition, textbook recommendations regarding stocking density of broilers may not be suitable in semi-arid tropical areas that are characterized by high ambient temperatures and humidity. The current study showed a linear decline in overall feed intake in response to increasing levels of stocking density. The birds raised under lower stocking densities (SD10 and SD20) consumed more feed and had higher weights compared to birds that were reared on higher stocking densities. These findings are similar to those reported by Thomas et al. [21] who observed that birds reared on low stocking density of 5 birds per m^2^, grew faster and consumed more feed than birds stocked at 10, 15, and 20 birds per m^2^. However, results in this study do not agree with those of Vargas-Rodriguez et al. [22] where all growth parameters were not affected by stocking density. Verheyen et al. [23] reported that as stocking density increases, feed intake decreases because physical access to feed and water is limited as the number of birds requiring feeder space increases. Thus, feed consumption decreases in birds reared under high stocking density resulting in poor FCE and weight gain. The effects are more pronounced as birds reach the grower and finisher phases when space availability becomes a major limiting factor [24]. This explains the current results were there was a positive quadratic response in weight gain observed only in weeks 5, but not earlier in the feeding trial.

Blood parameters are an effective, convenient tool for diagnosing and evaluating any pathophysiological abnormalities and nutritional status of animals [23]. Accordingly, haematological and serum biochemical indices were determined and used to monitor the effects of stocking density on the general welfare of the birds. Results from the present study indicated that there was neither linear nor quadratic trends for all haematological parameters in response to varying stocking densities. This contradicts Agusetyaningsih et al. [25] who found that a higher stocking density (16 birds/m^2^) resulted in higher levels of erythrocytes and haematocrits than a lower stocking density (10 birds/m^2^). In the current study, the concentration of serum glucose was higher in birds raised at lower stocking densities than in those reared at higher stocking densities. Since feed intake linearly declined in the current study, this can be explained by stocking density-induced changes in feed intake, which declined as density increased. Birds reared at lower stocking densities had higher serum glucose level because they consumed higher amounts of feed, translating to higher carbohydrate uptake, unlike birds reared at higher stocking densities whose feed intake was much lower.

The current serum glucose results are consistent with those reported by Karthiayini and Philomina [26], where serum glucose levels were reduced at high stocking density in broiler chickens. Indeed, the concentration of blood glucose is normally associated with environmental factors like heat stress and stocking density [27]. The current study showed that the serum concentration of albumin and ALT were higher in birds raised at a higher stocking density than in those reared at a lower stocking density. Nwaigwe et al. [28] and Jeong et al. [29] also showed that serum albumin concentrations increase in birds raised at a high stocking density. An increase in serum albumin is usually used as a reliable indicator of physiological stress in broilers [28]. Broilers tend to increase serum albumin as a homeostatic response to stress induced by high stocking density [30]. Alanine transaminase and AST are commonly used as effective biomarkers to identify tissue damages, especially in the liver. In the current study, birds reared at high stocking density (SD15, SD20, SD25, and SD30) had the highest levels of ALT and AST. Since broilers that are raised at high stocking densities endure increased competition for feed and water, the possibility of muscular injury is high and could be the reason for the higher levels of these two liver enzymes in the blood serum [31]. It was also observed that birds raised at the lowest stocking density (SD10) showed the lowest levels of SDMA. Usually, elevated levels of amino acid metabolites such as SDMA are harmful since this leads to oxidative stress, diseases, and disorders of the heart and neurological systems [32].

Carcass yields and weights of carcass portions are used when grading meat products, making them vital traits when determining sale prices [33]. In this study, SD20, SD25 and SD30 birds had lower final body weights compared to SD10 and SD15 birds. These results corroborated those reported by Thomas et al. [21] who observed that the average bird performance decreased when birds were reared at high stocking densities. Other carcass characteristics, which were negatively affected by stocking density include wing, drumstick, and duodenum weights. In contrast, Ravindran et al. [34] revealed that carcass characteristics were not affected by stocking density. These discrepancies between studies concerning the effect of stocking density on carcass characteristics of the broiler chickens may be explained by the different experimental conditions, such as bird strain [35], the presence or absence of antibiotics [34], or litter type [5]. Higher stocking densities have been reported to significantly decrease carcass yield and quality [36–40]. Current findings are in concordance with these observations, where carcass yield was higher at lower stocking densities but declined at higher stocking densities. Poor carcass yields at high stocking densities are mostly explained by the decrease in feed intake as the population of birds increases while feed space remains constant [1, 23]. Increasing stocking densities especially in the finisher period reduces physical access to feeders resulting in competition amongst the birds to get to the feeders. Verheyen et al. [23] reported a linear decline in feed intake as stocking density increased from 20 to 50 birds/m^2^, resulting in poor muscle development and fat deposition due to reduced nutrient uptake. Similar findings were reported by Dozier et al. [36], where increasing stocking density resulted in a linear reduction in absolute weight of the breast fillet. With regards to internal organs, jejunum and ilium weights linearly decreased as the stock density increased.

There are a limited number of studies that have investigated the effects of stocking density on intestinal development [41]. In this study, size of duodenum decreased with increasing stocking density, which agrees with the findings of Yin et al. [41] who reported similar results in geese. This means that high stocking density delays the general development of the small intestine, possibly due to lower feed intake and the associated nutrient deficiency. The weights of lymphoid organs like spleen are known to decrease in response to elevated levels of stress [42]. The same effect was observed in the current study where the weight of the spleen linearly decreased as the stocking the density increased. The bursa of *Fabricius* and spleen are crucial lymphoid organs in broiler chickens. Differentiation of B lymphocytes is completed in the bursa of *Fabricius* and the mature lymphocytes migrate to the peripheral lymphoid organs like the spleen [43]. Consequently, the reduction in the size of the spleen of broilers in the current study could have compromised the birds’ immune function. However, Heckert et al. [42] reported that stocking density had no influence on spleen weights. On the other hand, stress may also result in an increase in the weight of lymphoid organs to support immune responses in birds [44, 45].

The present study investigated the effect of stocking density on meat quality of Ross 308 broiler chickens. Stocking density did not affect any meat quality parameters observed. These findings are in accordance with previous studies [46–48] where stocking density did not induce any changes in meat quality traits. In contrast, Bilgili and Hess [49] stated that high stocking density compromises meat quality in broiler chickens. Genetic selection for rapid muscle growth in poultry has altered the ability of animals to respond and adapt to environmental stressors like stocking density [50]. Oxidative stress and tissue acidosis lead to cytotoxicity, free radical-mediated chain reaction, protein and lipid oxidation, impairment of animal health status and production resulting in products of inferior quality and shorter shelf life [51–53].

Stocking density had no influence on latency-to-lie of the birds. Various scholars have reported higher prevalence of hock and foot burns when birds are reared at high stocking densities [21, 54]. This has been attributed to high levels of moisture and ammonia trapped in the litter in highly stocked pens. Latency-to-lie test is used to assess the leg strength as a welfare indicator. The results were unexpected because leg strength was not significantly affected by stocking density. The current results are not consistent with Buijs et al. [55] who reported a decrease in leg strength with increasing density causing a decrease in LTL duration. Furthermore, other studies by Sørensen et al. [54] and Sanotra et al. [56] reported that broilers raised at high stocking densities show increased incidences of leg weaknesses.

## Conclusion

High stocking densities decreased overall feed intake, which negatively affected final body weight in Ross 308 broilers. An optimum stocking density of 20 birds/pen (~15 birds/m^2^ or 27.27 kg/m^2^) was determined in the current study, which is lower than the globally accepted density of 20 birds/m^2^. It can be recommended that feed additives with immune-boosting antioxidant and nutritional properties could be used if broiler chickens are reared at stocking densities higher than this optimum determined in the current study.

## Supporting information

S1 Table Chemical composition and digestible amino acids in starter, grower, and finisher diets used for feeding broilers. (PDF)
