# Supplementary material for: Stocking density-induced changes in growth performance, blood parameters, meat quality traits, and welfare of broiler chickens reared under semi-arid subtropical conditions
Source: PLoS One. 2022 Oct 13;17(10):e0275811. doi: 10.1371/journal.pone.0275811 (PMC9560488; doi:10.1371/journal.pone.0275811)
Supplement: S1 Table — (PDF) [file pone.0275811.s001.pdf]

S1 Table. Chemical composition and digestible amino acids in starter, grower, and finisher diets used for feeding broilers.

**Parameters**	**Starter phase**	**Grower phase**	**Finisher phase**
Metabolisable energy (MJ/Kg)	10.1	9.8	9.9
Organic matter (%)	82.0	82.2	82.6
Dry matter (%)	87.7	86.6	86.6
Crude protein (%)	18.2	16.8	15.4
Crude fibre (%)	6.11	0.36	5.14
Crude fat (%)	3.26	3.36	3.42
Sodium (%)	0.14	0.14	0.14
Calcium (%)	0.84	0.73	0.66
Chloride (%)	0.25	0.25	0.25
Digestible methionine (%)	0.41	0.44	0.51
Digestible lysine (%)	0.76	0.85	0.98
Digestible tryptophan (%)	0.12	0.14	0.15
Digestible threonine (%)	0.54	0.58	0.68
Digestible isoleucine (%)	0.5	0.55	0.6
Digestible arginine (%)	0.75	0.83	0.91
